# Radiation Safety Renaissance: Will State Regulations Help or Hinder an Emergence From the Dark Ages?

**DOI:** 10.1016/j.jscai.2025.103612

**Published:** 2025-04-30

**Authors:** Ryan D. Madder

**Affiliations:** Department of Cardiovascular Medicine, William Beaumont University Hospital, Corewell Health East, Royal Oak, Michigan

The results of the recently published 2023 SCAI Survey evaluating occupational health hazards in the cardiac catheterization laboratory are sobering.^1^ Two-thirds of our colleagues experience work-related pain attributable to wearing lead aprons, 60% experience an orthopedic injury, and nearly 1 in 5 limit their procedural workload owing to such an injury. An astounding 11% of respondents acknowledged knowing a colleague with a brain tumor. These disturbing results, which unfortunately reflect a lack of significant progress in catheterization laboratory occupational safety since the 2014 SCAI survey,^2^ fit uncomfortably well with a growing body of literature associating our profession with increased risks of orthopedic injuries, premature cataracts, carotid atherosclerosis, cognitive decline, DNA damage, and malignancies.

But, there is reason for hope. The growing recognition of the untenable occupational hazards has recently spawned the development of a multitude of innovative technologies having the potential to radically transform catheterization laboratory radiation safety practices. Owing to the emergence of these enhanced radiation protection devices (ERPDs), the field of interventional cardiology is on the precipice of a radiation safety renaissance. Early studies with some of these novel ERPDs have shown it is now possible to reduce radiation exposure among physicians and staff members to such low levels that wearing lead aprons may be unnecessary.^3^, ^4^, ^5^, ^6^, ^7^, ^8^, ^9^ Even if the first generation of some ERPDs do not quite achieve zero radiation exposure, it is likely that further refinements of these devices will, thereby making lead aprons obsolete. Liberating physicians and staff members from the burden of lead aprons would intuitively reduce the rates of the work-related pain and orthopedic injuries that have historically plagued our profession, but will regulatory agencies allow us to remove our lead?

In this issue of *JSCAI*, Vora et al^10^ present various details of radiation safety regulations pertaining to catheterization laboratories in the United States. As noted by the authors, facilities performing fluoroscopic imaging in the United States are regulated at the state level, thus creating a situation wherein 50 different state regulatory agencies govern radiation safety in catheterization laboratories across the United States. In the face of this heterogeneous and potentially confusing governance structure, the document by Vora et al^10^ serves as an excellent resource for physicians, catheterization laboratory staff members, trainees, and administrators to easily identify the state agency and associated regulations that oversee radiation safety in their catheterization laboratories. The comprehensive document by Vora et al^10^ is particularly timely given the emergence of ERPDs.

There is a lack of consistency among states in many radiation safety regulations, and these inconsistencies have implications for ERPD adoption. For instance, several states specifically mandate the use of lead aprons; some of them require lead aprons of 0.25 mm Pb, whereas others require lead aprons of 0.5 mm Pb. Considering a major impetus to purchase an ERPD is to facilitate either a lead-free environment or the use of lighter lead aprons, the existing regulations in many states have the potential to impede ERPD uptake and the associated doffing of lead aprons, thereby perpetuating the high rates of work-related pain and orthopedic injuries commonplace in our profession.

Although state regulatory agencies may hamper the development of safer work environments by failing to modernize their existing regulations in the era of ERPD, it is also possible that state regulations could evolve to accelerate radiation safety practices. There is widespread acceptance among both federal and state agencies that radiation doses should always be as low as reasonably achievable (ALARA). With ERPD, median radiation doses of 0.0 μSv among physicians and staff members are now reasonably achievable,^4^, ^5^, ^6^ thus signaling a paradigm shift in radiation doses consistent with the ALARA principle in contemporary practice. State regulations, if intent on maximizing workplace safety and minimizing the risk of occupational injury, should be updated to reflect the new ALARA. This could be achieved if states developed regulations to mandate the use of ERPD rather than lead aprons and encouraged other progressive safety measures such as consistent use of real-time dosimetry. Such updates in state regulations might help overcome the lack of buy-in among hospital administrators recently reported to be a significant barrier to ERPD uptake.^1^ If regulations do not evolve in parallel with the ongoing advances in ERPD technology, then state agencies will hinder a departure from the status quo in which catheterization laboratories are simply not a safe place to work.
